# Activation of Peripheral Blood CD4+ T-Cells in IBS is not Associated with Gastrointestinal or Psychological Symptoms

**DOI:** 10.1038/s41598-019-40124-5

**Published:** 2019-03-06

**Authors:** Yasmin Nasser, Carlene Petes, Celine Simmers, Lilian Basso, Christophe Altier, Katrina Gee, Stephen J. Vanner

**Affiliations:** 1 Gastrointestinal Diseases Research Unit, Kingston General Hospital, Queen’s University, Kingston, Ontario, Canada; 2 Department of Biomedical and Molecular Sciences, Queen’s University, Kingston, Ontario, Canada; 3 Department of Physiology and Pharmacology, Inflammation Research Network, Snyder Institute for Chronic Diseases, Cumming School of Medicine, University of Calgary, Calgary, Alberta Canada; 4 Present Address: Division of Gastroenterology and Hepatology, Department of Medicine, Cumming School of Medicine, University of Calgary, Health Sciences 1667, 3330 Hospital Drive NW, Calgary, Alberta T2N 4N1 Canada

## Abstract

Immune activation may underlie the pathogenesis of irritable bowel syndrome (IBS), but the evidence is conflicting. We examined whether peripheral CD4+ T-cells from IBS patients demonstrated immune activation and changes in cytokine production. To gain mechanistic insight, we examined whether immune activation correlated with psychological stress and changing symptoms over time. IBS patients (n = 29) and healthy volunteers (HV; n = 29) completed symptom and psychological questionnaires. IBS patients had a significant increase in CD4+ T-cells expressing the gut homing marker integrin β7 (p = 0.023) and lymphoid marker CD62L (p = 0.026) compared to HV. Furthermore, phytohaemagglutinin stimulated CD4+ T-cells from IBS-D patients demonstrated increased TNFα secretion when compared to HV (p = 0.044). Increased psychological scores in IBS did not correlate with TNFα production, while stress hormones inhibited cytokine secretion from CD4+ T-cells of HV *in vitro*. IBS symptoms, but not markers of immune activation, decreased over time. CD4+ T-cells from IBS-D patients exhibit immune activation, but this did not appear to correlate with psychological stress measurements or changing symptoms over time. This could suggest that immune activation is a surrogate of an initial trigger and/or ongoing parallel peripheral mechanisms.

## Introduction

Irritable bowel syndrome (IBS) is typified by chronic abdominal pain associated with a change in stool form and frequency^1^. It is a common and heterogeneous condition, and patients can be further subtyped based on their predominant bowel habit. To date, the pathogenesis of IBS is poorly understood, but is thought to involve an interplay between central and peripheral mechanisms^2–7^. As one of the strongest risk factors for developing IBS is a prior gastroenteritis, low-grade immune activation has been hypothesized to play a key role in the pathophysiology of IBS^8–13^. Low-grade immune activation could also be the result of increased physiological stress in IBS^2,3,5^. Indeed, changes at the level of both the adaptive and the innate immune system have been observed both in biopsy and peripheral blood studies of IBS patients and these could contribute to visceral pain through immune-mediated sensitization of afferent nerves^14–16^, or through potentiation of central symptoms as suggested in comorbid psychiatric disease^4,6,7^.

One of the challenges in IBS research has been the lack of a biomarker that identifies this disorder. Given the role of immune activation in IBS, there has been considerable interest in examining serum cytokine profiles. Unfortunately, there is a lack of consistent data with regards to the concentration of serum and plasma cytokines in IBS^2,3,10,12,17,18^. This inconsistency may be secondary to the multifactorial aetiology of IBS, the heterogeneity of IBS patients, or the lack of examination of specific immune cell types, thus resulting in signal dilution. Inherently, examining cytokines secreted from a specific cell type should enhance sensitivity, although this approach is more laborious than simply measuring serum cytokines, requiring increased technical expertise and extended studies over time. Furthermore, although studies have examined immune activation at a particular time point, longitudinal studies have been lacking, despite the fact that IBS is a chronic disorder and immune activation could play a role in sustaining symptoms.

The adaptive immune system is important in perpetuating and sustaining symptoms^4^. In particular, CD4+ T-cells are known to play a key role in visceral pain and hypersensitivity^19^. Colonic mucosal biopsies from IBS patients demonstrate increased infiltration of the lamina propria by CD4+ T-cells^8,9,13^ and an increased frequency of activated CD4+ T-cells is seen in the peripheral blood in IBS patients^20^. Furthermore, there is an increase in gut-homing T-cells^11,20^ in the peripheral blood in IBS, suggesting increased adaptive immune activation. Despite the evidence for an increase in activated CD4+ T-cells in IBS, there is little data on CD4+ T-cell derived cytokines.

Our aim was to examine markers of the adaptive immune system to determine its potential role in the expression of IBS. We first sought to determine whether there was evidence of immune activation in IBS, by examining peripheral blood CD4+ T-cell homing and stimulated cytokine production as a sensitive assay of immune activation. To gain mechanistic insights, we assessed whether CD4+ T-cell immune activation could be related to signalling from the central nervous system, by examining the relationship between CD4+ T-cell activation, chronic gut symptoms, psychological distress and stress hormones. Furthermore, we correlated changes in symptoms over time with cytokines released by stimulated peripheral CD4+ T-cells. We chose to concentrate on TNFα, IL-6, and IL-10 since previous reports have demonstrated evidence for the existence of TNFα, IL-6 and IL-10 gene polymorphisms in IBS^21,22^, and these cytokines have been well studied in the serum^2,3,10,12,17,18^ and peripheral blood mononuclear (PBMC) populations^16,23–28^ in IBS.

## Results

### Study subjects

A total of 29 healthy volunteers (HV) and 29 IBS patients were recruited. Of these, 10 were classified as IBS with diarrhoea predominance (IBS-D; 34%), 11 as IBS with constipation predominance (IBS-C; 38%), and 8 as IBS with mixed bowel habits (IBS-M; 28%). No differences in age or sex were noted when comparing IBS patients vs. HV (Table 1). The mean BMI of IBS patients was significantly elevated when compared to HV (p = 0.0042). IBS patients had significantly worse quality of life (QOL) (p < 0.0001) and higher symptom severity scores (IBS-SSS), which were in the moderately severe range^29^, when compared to HV (p < 0.0001).

**Table 1:** Baseline characteristics of patients and healthy volunteers.

	HV (n = 29)	IBS (n = 29)	IBS-D (n = 10)	IBS-C (n = 11)	IBS-M (n = 8)
Age (years)	47.9 ± 2.0	50.7 ± 3.3	37.8 ± 3.9	62.8 ± 4.3^#^	50.0 ± 6.4
Female Sex	89.7%	82.8%	70.0%	100%	75%
BMI	24.2 ± 0.6	28.4 ± 1.5**	28.3 ± 3.5	27.4 ± 2.0	29.6 ± 2.3
IBS-SSS	12.5 (0–30.0)	257.0 (210.0–352.3)***	286.5 (241.0–371.0)^&^	284.0 (235.0–355.0)^&^	197.0 (146.3–258.5)^$^
IBS-QOL	0 (0–3.0)	103.3 (74.9–137.0)***	112.0 (92.0–154.5)^&^	93.0 (35.0–134.0)^&^	109.0 (69.0–133.8)^&^

HV: Healthy Volunteers. IBS: Irritable bowel syndrome. IBS-D: diarrhoea-predominant IBS, IBS-C: constipation-predominant IBS, IBS-M: IBS with mixed symptoms. IBS-SSS: IBS Symptom Severity Score. IBS-QOL: IBS-36 Quality of Life. BMI: Body mass index. Age and BMI data expressed as Mean ± SEM. **p = 0.0042 IBS vs. HV, ***p < 0.0001 IBS vs. HV. Note: for IBS-QOL, n = 28 only for IBS (n = 9 for IBS-D). Subgroup analyses: ^#^p = 0.0005 ANOVA, p < 0.001 IBS-D vs. IBS-C. ^&^p < 0.0001 Kruskal Wallis test, p < 0.001 vs. HV and ^$^p < 0.01 vs. HV.

With regards to IBS subgroups, IBS-C patients were significantly older than IBS-D patients (p < 0.001). No differences in sex, BMI, IBS-SSS, or QOL scores were noted between subgroups; all IBS subgroups had a significantly elevated symptom severity score (p < 0.001 for IBS-D and IBS-C vs. HV; p < 0.01 IBS-M vs. HV) and lower quality of life (p < 0.001 for IBS-D, IBS-C and IBS-M vs. HV) when compared to HV. No significant differences in the use of NSAIDs or PPIs were noted between IBS subgroups (data not shown).

Demographic data of patients in the flow cytometry and longitudinal components of the study are presented in Table 2. Similar to the main cohort, no differences in age or sex were detected between patients and HV; the mean BMI of IBS patients was also significantly increased when compared to controls for the flow cytometry analysis (p = 0.0030). For the longitudinal component, no differences in sex (p = 0.08) or BMI (p = 0.22) were noted between IBS-D and HV.

**Table 2:** Baseline characteristics of patients and healthy volunteers in the flow cytometry and longitudinal components of the study.

	Flow Cytometry		Longitudinal	
	HV (n = 12)	IBS (n = 12)	HV (n = 15)	IBS-D (n = 7)
Age (years)	50.6 ± 2.3	46.3 ± 4.1	49.2 ± 1.9	40.7 ± 5.1
Female Sex	83.3%	75.0%	93.3%	57.1%
BMI	25.1 ± 0.9	33.2 ± 1.8**	24.4 ± 0.7	29.8 ± 3.9
IBS-SSS	12.5 (0–29.3)	237.0 (195.5–342.0)***	13.0 (0–30.0)	306.0 (229.0–389.0)^$^
IBS-QOL	0.4 (0–4.5)	113.5 (69.8–130.8)***	0 (0–1.6)	112.0 (81.0–150.0)^#^

HV: Healthy Volunteers. IBS: Irritable bowel syndrome. IBS-D: diarrhoea-predominant IBS, IBS-SSS: IBS Symptom Severity Score. IBS-QOL: IBS-36 Quality of Life. BMI: Body mass index. Age and BMI data expressed as Mean ± SEM **p = 0.0030 vs. HV, ^$^p = 0.0002 vs. HV, ^#^p = 0.0001 vs. HV and ***p < 0.0001 vs. HV.

### Increased gut homing CD4+ T-cells are noted in IBS, suggesting immune activation *in vivo*

**Figure 1:**
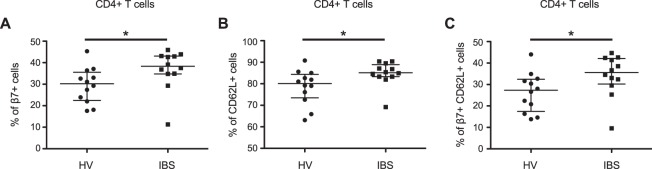
Flow cytometry for integrin β7 and CD62L in IBS patients and HV. (**A**) Flow cytometry revealed a significant increase in the % of CD4+ T-cells expressing the gut homing marker, integrin β7, in patients with IBS (38.3% [34.8–43.1], n = 12) when compared to HV (30.2% [22.5–35.6], n = 12; p = 0.023 vs. IBS). A significant increase in the % of CD4+ T-cells expressing the lymph node homing marker, CD62L was also seen in IBS (**B**) (IBS: 85.2% [83.3–88.9], n = 12; HV: 80.1% [73.5–84.4], n = 12; p = 0.026), as well as in the % of CD4+ T-cells co-expressing CD62L and integrin β7 (**C**) (IBS: 35.6% [30.2–42.2], n = 12; HV: 27.3% [17.4–32.5], n = 12; p = 0.040, when compared to HV).

We first examined whether there was evidence of immune activation *in vivo* in IBS. We performed flow cytometry to phenotype CD4+ T-cells and examined the expression of the gut mucosal homing marker integrin β7^30,31^ and the lymph node homing marker CD62L^32^. We examined the CD4+ T-cell population from the total PBMC population, rather than isolated CD4+ T-cells, as the PBMC population more closely resembles the population in circulation *in vivo* and because these cells had been least subjected to manipulation, since this may alter the expression of cell surface markers such as integrins^33,34^. Flow cytometry revealed a significant increase in the percentage of CD4+ T-cells expressing integrin β7 (p = 0.023 vs. IBS; Fig. 1A), and CD62L (p = 0.026; Fig. 1B) in IBS patients when compared to HV. CD4+ T-cells co-expressing CD62L and integrin β7 were also significantly increased in patients with IBS when compared to HV (p = 0.040; Fig. 1C).

### CD4+ T-cell stimulated TNFα is elevated in patients with IBS-D

**Figure 2:**
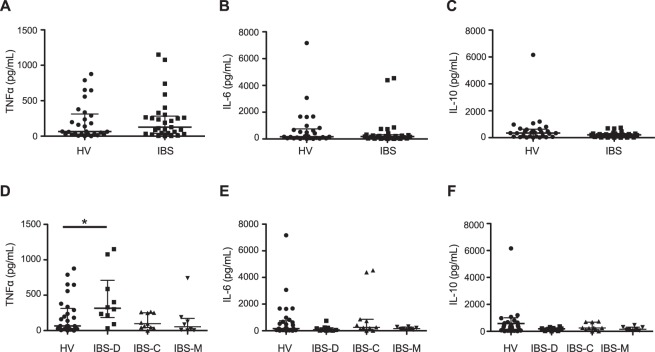
CD4+ T-cell stimulated cytokines in IBS patients and HV. No differences in CD4+ T-cell stimulated TNFα (**A**), IL-6 (**B**) or IL-10 (**C**) were seen when comparing HV (n = 29) to IBS patients (n = 29). CD4+ T-cell derived TNFα concentrations were significantly increased in IBS-D (315.3 pg/mL [184.3–710.3], n = 10) patients (**D**) when compared to HV (65.2 pg/mL [28.6–313.6], n = 29. p = 0.044 Kruskal Wallis Test, p < 0.05 HV vs. IBS-D). No significant differences were seen in CD4+ T-cell derived IL-6 (**E**) or IL-10 (**F**) concentrations when comparing IBS subgroups.

As we observed increased gut and lymphocyte homing of CD4+ T-cells, we next examined whether CD4+ T-cell stimulated cytokines were increased, as further evidence of immune activation in IBS. Unstimulated CD4+ T-cell derived TNFα, IL-6, and IL-10 were undetectable in the majority of patients and were not significantly elevated in the few patients who demonstrated baseline cytokine expression (data not shown). PHA stimulation of CD4+ T-cells increased the concentration of all three cytokines, as expected^35,36^. Stimulated CD4+ T-cell derived TNFα (Fig. 2A), IL-6 (Fig. 2B) and IL-10 (Fig. 2C) were not changed in IBS patients when compared with HV. However, a significant increase in CD4+ T-cell derived TNFα was observed in patients with IBS-D when compared to HV (Fig. 2D. Kruskal Wallis Test p = 0.044; HV vs. IBS-D p < 0.05). No change in CD4+ T-cell derived IL-6 (Fig. 2E) or IL-10 (Fig. 2F) was observed when comparing IBS subgroups. Since the mean BMI of IBS patients was significantly elevated and as visceral adipose tissue has been shown to be a source of circulating TNFα^37^, we tested whether there was a correlation between BMI and CD4+ T-cell derived TNFα. TNFα concentrations were not correlated with BMI (Spearman r = 0.03; p = 0.81).

### Serum cytokine concentrations are largely undetectable in both IBS patients and HV

In contrast, serum IL-6, IL-10 and TNFα were largely undetectable in both IBS patients and HV using ELISA; no differences were seen when comparing IBS patients and HV, nor were differences between IBS subgroups noted (Table 3). In order to verify that this was not a methodological issue, a Luminex assay was also performed. Although low concentrations of TNFα were detected in IBS patients and HV with the Luminex assay, no differences were seen when comparing IBS and HV or between IBS patient subgroups; IL-6 and IL-10 were largely undetectable, and no differences were observed between groups (data not shown), suggesting that serum cytokines are less sensitive to detect immune activation in IBS.

**Table 3:** Serum cytokines in patients and healthy volunteers.

	HV (n = 29)	IBS (n = 29)	IBS-D (n = 10)	IBS-C (n = 11)	IBS-M (n = 8)
TNFα (pg/mL)	0 (0–0)	0 (0–0)	0 (0–0)	0 (0–0)	0 (0–0)
IL-6 (pg/mL)	0 (0–0)	0 (0–0)	0 (0–0)	0 (0–0)	0 (0–5.9)
IL-10 (pg/mL)	4.7 (0.2–7.1)	1.4 (0–8.1)	0.8 (0–10.3)	3.5 (0–6.8)	0 (0–9.5)

HV: Healthy Volunteers. IBS: Irritable bowel syndrome. IBS-D: diarrhoea-predominant IBS, IBS-C: constipation-predominant IBS, IBS-M: IBS with mixed symptoms. No significant differences between HV and IBS patients, or between IBS subgroups were seen.

### T-cell derived cytokines are not related to psychological or symptom severity scores

**Figure 3:**
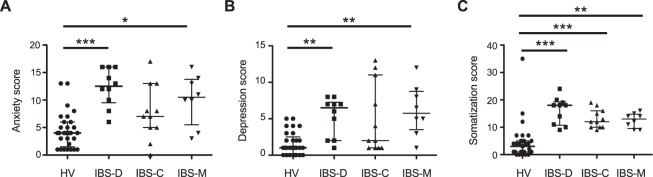
Psychological scores in IBS patients and HV. Anxiety and depression scores were assessed using the anxiety and depression components of the HADS score, while somatization was assessed using the PHQ-15 score. The anxiety (**A**) (HV: 4.0 [1.5–6.0], n = 29; IBS-D: 12.5 [9.5–16.0], n = 10; IBS-C: 7.0 [5.0–13.0], n = 11; IBS-M: 10.5 [5.5–13.8], n = 8; Kruskal Wallis Test p < 0.0001; HV vs. IBS-D p < 0.001, HV vs. IBS-M p < 0.05) and depression (**B**) (HV: 1.0 [0–2.5], n = 29; IBS-D: 6.5 [2.0–7.3], n = 10; IBS-C: 2.0 [1.0–11.0], n = 11; IBS-M: 5/8 [3.5–8.8], n = 8; Kruskal Wallis Test p < 0.0001; HV vs. IBS-D p < 0.01, HV vs. IBS-M p < 0.01) scores for both IBS-D and IBS-M patients were significantly different from HV; no differences between subgroups was noted. Similarly, while all IBS subtypes exhibited increased somatization scores when compared to HV (**C**) (HV: 3.0 [1.0–5.0], n = 29; IBS-D: 18.0 [10.8–19.3], n = 10; IBS-C: 12.0 [10.0–16.0], n = 11; IBS-M: 13.0 [9.5–14.8], n = 8; Kruskal Wallis Test p < 0.0001; HV vs. IBS-D p < 0.001, HV vs. IBS-C p < 0.001, HV vs. IBS-M p < 0.01), no differences between subgroups were noted.

In order to elucidate the mechanism for the elevation in CD4+ T-cell stimulated cytokine secretion, we examined whether there was a relationship between TNFα and the severity of IBS symptoms or comorbid psychological conditions, such as anxiety, depression and somatization in IBS subgroups^5^. IBS-D and IBS-M patients displayed increased anxiety (Fig. 3A, Kruskal Wallis Test p < 0.0001; HV vs. IBS-D p < 0.001, HV vs. IBS-M p < 0.05) and depression (Fig. 3B, Kruskal Wallis Test p < 0.0001; HV vs. IBS-D p < 0.01, HV vs. IBS-M p < 0.01), while all three subtypes displayed increased somatization (Fig. 3C, Kruskal Wallis Test p < 0.0001; HV vs. IBS-D p < 0.001, HV vs. IBS-M p < 0.01, HV vs. IBS-C p < 0.001) when compared to HV. However, no significant differences in psychological scores were seen when the IBS subgroups were compared to each other.

As we saw significant differences in CD4+ T-cell derived TNFα, correlation analyses between psychological and symptom severity scores were only performed with TNFα. TNFα concentrations were not correlated with the severity of anxiety (Spearman r = 0.15; p = 0.27), depression (Spearman r = 0.18, p = 0.19) or somatization (Spearman r = 0.14; p = 0.28), suggesting that psychological symptoms and CD4+ T-cell stimulated cytokines were not related. No significant correlation between TNFα and IBS symptom severity (Spearman r = 0.11; p = 0.40) or quality of life (Spearman r = 0.15; p = 0.28) was noted.

### Stimulated cytokines do not change over time, despite an improvement in somatization and symptom severity scores in IBS patients

Given the paucity of data with regards to immune activation in IBS and how this might correlate with changing symptoms over time, we recruited IBS-D patients and HV to return to the clinic 3–5 months after their initial visit. We chose to solely examine IBS-D patients since we had seen an increase in CD4+ T-cell derived TNFα in these patients. PHA-stimulated CD4+ T-cell derived TNFα (Table 4), IL-6 and IL-10 (data not shown) were unchanged over time in IBS-D patients when compared to HV. Although anxiety and depression scores were unchanged, a marked decrease in the somatization score (Table 4, p = 0.0012) was noted over the course of the study when comparing IBS patients to HV. Similarly, a significant improvement in IBS symptom severity (Table 4, p = 0.0098) was seen over time in IBS-D patients; QOL scores were unchanged (Table 4) when comparing IBS-D patients to HV. However, the change in stimulated CD4+ T-cell derived TNFα was not significantly correlated with the change in psychological scores, symptom severity, or QOL (data not shown).

**Table 4:** Stimulated cytokines and psychological/symptom scores over time in patients and healthy volunteers.

	HV (n = 15) Initial visit	HV (n = 15) Return visit	IBS-D (n = 7) Initial visit	IBS-D (n = 7) Return visit
TNFα (pg/mL)	29.4 (12.9–138.2)	67.3 (23.7–118.8)	301.3 (90.1–588.1)	355.4 (69.5–614.9)
Anxiety	4.0 (3.8–5.3)	3.0 (2.0–6.3)	12.0 (8.0–16.0)	11.0 (4.0–14.0)
Depression	1.0 (0.8–2.3)	1.5 (0–3.0)	6.0 (2.0–8.0)	5.0 (4.0–8.0)
PHQ15	3.5 (1.0–4.3)	4.5 (2.8–8.0)	18 (11.0–20.0)	12 (10.0–19.0)*
IBS-SSS	12.8 (0–27.8)	18.3 (4.5–37.0)	306.0 (229.0–389.0)	265.5 (218.0–300.0)^$^
IBS-QOL	0 (0–2.5)	3.0 (0–6.3)	112.0 (81.0–150.0)	100.0 (48.0–145.0)

HV: Healthy Volunteers. IBS-D: diarrhoea-predominant IBS, IBS-C: constipation-predominant IBS, IBS-M: IBS with mixed symptoms. PHQ15: Somatization score, IBS-SSS: IBS Symptom Severity Score. IBS-QOL: IBS-36 Quality of Life score. Note: for Anxiety, depression, PHQ15, IBS-SSS and IBS-QOL scores, n = 14 for HV. Raw data is shown. The change in cytokine concentration or symptom score was calculated by subtracting the initial measurement or score from the repeat and analysed. *p = 0.0012 vs HV; ^$^p = 0.0098 vs HV.

### Stress hormones decrease CD4+ T-cell cytokine secretion

**Figure 4:**
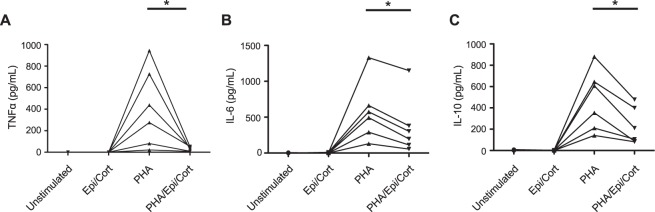
The effect of stress hormones on CD4+ T-cell stimulated cytokine production. CD4+ T-cells were incubated with media alone, PHA 5 μM and/or the stress hormones epinephrine 1 nM + corticosterone 1 μM. Epinephrine and corticosterone significantly inhibited CD4+ T-cell stimulated production of TNFα (**A**) [Unstimulated: 0 pg/mL (0–0); Epinephrine/corticosterone alone: 0 pg/mL (0–0); PHA: 356.6 pg/mL (65.6–780.6); PHA + Epinephrine/corticosterone: 34.6 pg/mL (8.8–51.3)], IL-6 (**B**) [(Unstimulated: 0.4 pg/mL (0–3.5); Epinephrine/corticosterone alone: 0.7 pg/mL (0.2–7.7); PHA: 533.8 pg/mL (250.9–830.1); PHA + Epinephrine/corticosterone: 250.3 pg/mL (98.5–571.6)] and IL-10 (**C**) [(Unstimulated: 1.1 pg/mL (0.7–5.3); Epinephrine/corticosterone alone: 1.0 pg/mL (0.02–3.8); PHA: 481.0 pg/mL (192.9–703.3); PHA + Epinephrine/corticosterone: 158.3 pg/mL (86.7–419.1)]. Individual responses from each patient are shown graphically. *p = 0.031 vs. PHA response; n = 6 donors in all cases.

Previous studies have suggested that psychological stress can cause immune activation^4^; however stress hormones are also known to suppress immune activation^38–40^. Therefore, we tested whether epinephrine and corticosterone could play a role in the increased CD4+ T-cell stimulated cytokines. To this end, isolated CD4+ T-cells from HV were stimulated with PHA, with or without the addition of epinephrine and corticosterone. In unstimulated CD4+ T-cells as well as cells incubated with epinephrine and corticosterone alone, TNFα, IL-6, and IL-10 were minimally present. PHA stimulation resulted in increased TNFα (Fig. 4A), IL-6 (Fig. 4B) and IL-10 (Fig. 4C) secretion. The addition of epinephrine and corticosterone abrogated PHA-induced TNFα secretion while partially inhibiting IL-6 and IL-10 production. Taken together, this data demonstrate that stress hormones downregulate PHA-induced CD4+ T-cell cytokine production *in vitro*.

## Discussion

This study examined whether immune activation is present in IBS and if so, whether it is mechanistically linked to factors underlying psychological distress. We found that IBS patients had evidence of immune activation with an increased percentage of CD4+ T-cells expressing the gut homing marker integrin β7 and the lymph node homing marker CD62L, while CD4+ T-cell derived TNFα was increased in patients with IBS-D when compared to HV. In contrast, serum cytokines were comparable. GI and psychological symptoms, but not immune activation, improved over time in IBS patients, potentially suggesting differing underlying mechanisms.

We observed a significant increase in CD4+ T-cells expressing the gut homing marker integrin β7 and the lymph node homing marker CD62L in IBS; cells co-expressing CD62L and integrin β7 were also significantly increased in IBS. CD62L is integral for mediating T-cell rolling on the endothelium of peripheral lymph nodes as well as mucosal associated lymphoid tissues, while integrin α4β7 specifically mediates T-cell trafficking into gut associated lymphoid tissue^31,32^. In the current study, we did not further differentiate between memory and naïve subpopulations of CD4+ T-cells, which can both express CD62L^31,32^. Our data supports previous studies which demonstrate enhanced integrin β7 expression on T-cells in patients with IBS and functional dyspepsia^11,20,41^, although not all studies have been positive^27^. Taken together, our data and that from previous studies suggest that there are changes in lymphocyte homing in functional bowel disorders, which may indicate immune activation. Further studies are needed to determine whether this plays a causative role in IBS.

We demonstrated that serum cytokines were largely undetectable in IBS patients, whereas PHA-stimulated CD4+ derived T-cell cytokine production was detectable, suggesting that cytokine release from a particular cell subtype is a more sensitive means to determine cell-mediated immune responses to antigenic stimulation^23^. As such, examining stimulated CD4+ T-cell secreted cytokine production from IBS patients compared to HV may reveal evidence of differential immune activation that was not apparent by solely examining serum cytokine concentrations. This may explain, at least in part, the mixed results in the literature, as serum/plasma concentrations of TNFα have been found to be unchanged^2,3,12^, increased^10,18^ or decreased^26^ in IBS patients compared to controls. Elevated CD4+ derived T-cell TNFα suggests that IBS-D patients may have a hyperresponsive immune system in response to stimulation^12,20^, since little baseline TNFα was seen in serum or unstimulated cells. Interestingly, the BMI of IBS patients in our study was significantly elevated when compared to controls. Visceral adipose tissue is known to be a source of circulating TNFα^37^; however we did not observe differences in BMI between IBS subgroups, nor did we find a correlation between TNFα and BMI in our study. In agreement with our data, an increase in PBMC-derived TNFα was observed in IBS-D but not IBS-C patients^16,23^, while others have seen an increase in PBMC-derived TNFα in all IBS patients, without examining IBS subtypes^25^. However, this has not been a universal finding in IBS and some of this variation may be explained by methodological differences^27,28^ or patient characteristics. Thus, it would appear that TNFα secretion from CD4+ T-cells in IBS may be a more sensitive assay of immune activation, when compared to serum cytokines.

Previous studies have found that only a subset of IBS patients are “immune-active” patients^42–44^. Our data demonstrate overlap of TNFα concentrations between IBS-D patients and HV. Further studies are required to define whether stimulated cytokine cut-offs can categorize patients as “immune-active”^43^.

There is extensive data on the bi-directional interaction between the immune system and psychological symptoms/physiological stress^4^. We wanted to determine whether the observed increased in CD4+ T-cell derived TNFα was correlated with psychological symptoms in IBS or was related to stress hormones. In the present study, we demonstrated that patients with IBS had significantly increased psychological scores when compared to HV, in agreement with previous studies^6,23,28^. Pro-inflammatory cytokines activate the hypothalamic pituitary adrenal (HPA) axis^25^, which is known to be dysregulated in IBS^2,5,7,45,46^. Psychological scores were similar between IBS subtypes in our study, while increased CD4+ T-cell derived TNFα was only seen in the IBS-D subtype, suggesting that stress, psychiatric symptoms and cytokine secretion were not strongly linked. In agreement with this, we did not find a correlation between the severity of psychological symptoms and CD4+ T-cell stimulated TNFα secretion. Furthermore, corticosterone and epinephrine, at similar concentrations to those found in the sera of IBS patients^2,5,7^, induced a decrease in cytokine secretion from PHA-stimulated CD4+ T-cells of HV *in vitro*. This is in agreement with studies from animal models, where stress hormones suppressed TNFα production and the mitogenic response of CD4+ T-cells in a dose dependent manner^38–40^. Our data was surprising, as previous studies have shown a positive correlation between stimulated TNFα secretion in functional GI disorders with the anxiety^23,28^ and depression^28^ components of the HADS score. However, a more recent report also noted a disconnect between serum cytokines and symptom characteristics in IBS^43^. We cannot however exclude whether local immune responses largely confined to the intestine (e.g. mast cell activation) are correlated with symptom characteristics. Future studies in a larger cohort of IBS patients should examine whether there is also a disconnect between systemic and local immune activation and the severity of IBS symptoms.

To determine whether immune activation in IBS-D patients was correlated with changing IBS symptoms, we conducted a longitudinal study in a small cohort. We found that while immune activation was unchanged over time in IBS-D patients, symptom severity and somatization scores improved at the follow up visit, further supporting a potential dissociation between low-grade immune activation and psychiatric symptom/gut symptom severity in IBS. Our finding of improving symptom severity and somatization scores in IBS-D may be reflective of an inadvertent “therapeutic alliance” in our population – by simply recalling our patients back for the study and paying increased attention to their symptoms, this subsequently resulted in improved symptom and psychiatric severity scores. This phenomenon has previously been demonstrated in IBS^47,48^. Since this “therapeutic alliance” is primarily a centrally acting phenomenon, it is unlikely to correlate with peripheral mechanisms such as low-grade immune activation. However, it should be noted that we did not control for environmental changes, alcohol consumption, probiotic use etc. in our small patient or HV cohort over time. A larger number of patients are needed to determine if a correlation exists over time in a subset of “immune-active” patients. Future studies should follow a larger cohort of patients longitudinally for an extended period of time, given the chronic nature of IBS, and correlate changes in immune activation and symptom severity with peripheral-acting mechanisms in IBS, such as changes in intestinal microbiota and intestinal permeability.

Systemic immune activation could lead to worsening central symptoms in IBS, through the potentiation of stress/HPA axis activation and psychological symptoms^4^. However, our data demonstrates a disconnect between central symptoms, such as anxiety, depression, somatization and CD4+ T-cell activation. Further, we did not observe a major correlation between CD4+ T-cell cytokine activation and the severity of gut symptoms, suggesting that these may not be directly related. Activation of CD4+ T-cells may be reflective of a previous trigger in IBS, or the result of peripheral mechanisms, such as changes in the microbiome or changes in intestinal permeability^14^. Further studies will be needed to elucidate the cause of CD4+ T-cell immune activation and whether this is related to the pathophysiology of IBS.

In conclusion, we have demonstrated that examination of CD4+ T-cell cytokines and gut homing reveals immune activation in IBS and that this seems largely confined to IBS-D patients. Immune activation did not correlate with changing symptoms over time or measures of psychological stress, suggesting it may be either a surrogate of a previous triggers and/or parallel peripheral mechanisms. Future studies will be needed to carefully examine this hypothesis.

## Methods

### Patients

Consecutive IBS patients (n = 29) and HV with no prior history of GI disorders or current GI symptoms (n = 29) were recruited from the outpatient gastroenterology clinic at the Hotel Dieu Hospital between November 2014- September 2015 (Kingston, ON, Canada). In addition, IBS-D patients (n = 7) and HV (n = 15) were recruited to participate in a longitudinal component of the study, which occurred 3–5 months after the initial study visit, at the time of a subsequent GI clinic follow up. For experiments involving stress hormones, healthy donors with no prior history of GI disorders or current GI symptoms (n = 6) were recruited. Subjects with active infections were excluded from the study. The Queen’s University Human Ethics Committee approved the protocol and all patients gave informed written consent [REB file number #6004988]. All research was performed in accordance with Canadian Tri-Council guidelines governing the ethical conduct for research involving humans.

Baseline characteristics are presented in Table 1. IBS and IBS subtype diagnoses were made on the basis of the Rome III criteria^1^. All patients with diarrhoea predominance had negative celiac testing (negative TTG and/or negative duodenal biopsies). All but 3 IBS patients (1 with diarrhoea predominance, 2 with mixed bowel habits) had undergone endoscopic or radiologic (CT colon) evaluation at the time of the study; there was no evidence of macroscopic or microscopic (see below) inflammation on these studies. Endoscopic or radiologic studies were performed to rule out other aetiologies of GI symptoms, including colon cancer in those with a family history or change in bowel habits > age 50, or random biopsies to rule out microscopic colitis in patients with diarrhoea predominance. Only 1 patient with diarrhoea did not have random biopsies to rule out microscopic colitis. Patients who had a co-diagnosis of an inflammation related disease or the use of medications known to affect the immune system other than non-steroidal anti-inflammatories (NSAIDs) were excluded from the study; no other restrictions were placed on medication use. None of the IBS patients gave a history suggestive of post-infectious IBS.

Patients and HV filled out a series of validated questionnaires at the initial visit and at the longitudinal follow up visit, if applicable, in order to assess the severity of IBS as well as psychological symptoms^49^. These consisted of the IBS-Symptom Severity Index (IBS-SSS^29^), the IBS-36 Quality of Life questionnaire (IBS-QOL^50^), the Hospital Anxiety and Depression Scale (HADS^51^) and the PHQ-15 Somatization Scale (PHQ-15^52^). Separate investigators scored/recruited patients from those who performed subsequent experiments; investigators performing the experiments were blinded to subject diagnosis and questionnaire scores until flow cytometry and/or ELISA analyses were completed.

### Isolation and stimulation of CD4+ T-cells

Peripheral blood mononuclear cells (PBMCs) were isolated from 10 mL of whole blood samples as described^53^. Briefly, whole blood samples from IBS patients and HV were diluted with an equal volume of phosphate buffered saline (PBS) containing EDTA (1 mM) + 2% foetal bovine serum (FBS; Hyclone, GE Healthcare Life Sciences, Logan, UT) and layered over the density gradient separation media Lympholyte (Cedarlane Laboratories; Burlington, ON, Canada). The samples were centrifuged and 1 mL of 1:1 PBS diluted serum was stored at −80 °C. The average yield of PBMCs was 21.0 ± 1.2 × 10^6^ in HV and 24.5 ± 2.6 × 10^6^ in IBS patients. In some cases, PBMCs (2 × 10^6^ cells/mL) were stored in RPMI freezing media (Gibco, ThermoFisher, Burlington, ON) containing 20% FBS and 10% DMSO (BioShop Canada Inc; Burlington, ON) in 1 mL volume in cell culture cryogenic tubes and stored at −80 °C^54^ for use in flow cytometry experiments. At a later date, the full set of cells were thawed, cultured for 16 hours and subsequently utilized for flow cytometry at the same time for consistency in experimental procedures.

CD4+ T-cells were immediately isolated by immunomagnetic negative selection from PBMCs using the EasySep Human CD4+ T-cell enrichment kit as per the manufacturer’s instructions (StemCell Technologies; Vancouver, BC, Canada). Purified T-cells were immediately stimulated with phytohaemagglutinin (PHA) as described below for cytokine assessment. In some cases, CD4+ T-cells (2 × 10^6^ cells/mL) were stored in RPMI freezing media at −80 °C for assessment of cell purity; purity was confirmed by CD3/CD4 expression by flow cytometry and was determined to be >98% pure. The average yield of CD4+ T-cells was 6.0 ± 0.5 × 10^6^ in HV and 5.3 ± 0.5 × 10^6^ in IBS patients. Due to limitations in cell numbers collected per blood draw, not all assays could be performed on every sample. The number of samples used for each analysis is indicated in the figures.

Purified CD4+ T-cells (2 × 10^6^ per well) from fresh cell suspensions were immediately cultured for 24 hours in RPMI + 10% FBS in the presence or absence of PHA (Sigma Aldrich, Oakville, ON, Canada) at 5 μg/mL^35^ for cytokine assessment. For experiments involving stress hormones, epinephrine (1 nM; Sigma) and corticosterone (1 μM; Sigma) were also added to the culture media for 24 hours; these concentrations are similar to those found in the sera of IBS patients^2,5,7^. This time point was chosen in order to mimic chronic stress in an *in vitro* model^40,55^. Supernatants were stored at −80 °C until use.

### Flow cytometry

Flow cytometry was performed as previously described^54^. Briefly, PBMCs or purified unstimulated CD4+ T-cells from all participants were thawed simultaneously overnight in 24-well tissue culture plates in RPMI media with 10% foetal bovine serum^54^; this approach has been shown to allow recovery of integrin expression following freeze-thaw^56,57^. Cells were washed in PBS + 0.1% sodium azide and stained with fluorochrome-conjugated antibodies: CD4-PECy5, CD62L-FITC, β7-PE (both from eBioscience, San Diego, CA) and CD3-ECD (Beckman Coulter Inc., Mississauga, ON) or the appropriate isotype control: IgG1-FITC, IgG2a kappa-PE, IgG1-PECy5 [all from Beckman Coulter Inc]. After staining, cells were washed in PBS-azide and data were acquired using an Epics XL-MCL flow cytometer (Beckman Coulter) and analysed using FlowJo software (Ashland, OR). Although viability was not tested following freeze- thaw, gates were drawn based on forward and side scatter to eliminate data collected from debris and dead cells for PBMC samples. Subsequently, the lymphocyte population was gated for CD4+ cells and analysed for CD62L and β7 expression.

### ELISA

Culture supernatants or serum samples were used in ELISA analysis to quantify cytokine expression of TNFα, IL-6, and IL-10 as per the manufacturer’s instructions (eBioscience). The lower limit of detection for TNFα was 4 pg/mL while for IL-6 and IL-10, the lower limit of detection was 2 pg/mL. Samples were measured in duplicate wells. Absorbencies were measured with a BioTek ELx800 Microplate Reader (ThermoFisher Scientific).

### Luminex

Serum samples were analysed for cytokine expression using a multiplex assay with the MILLIPLEX human MAP mouse cytokine/chemokine panel (EMD Millipore, Billerica MA) on a Luminex xMAP multiplexing technology (Eve Technologies, Calgary, Alberta, Canada) as previously described^58^.

### Statistics

Statistical analysis was performed using GraphPad Prism 5.0 (GraphPad Software Inc., La Jolla CA); p < 0.05 was considered significant. The D’Agostino & Pearson test was used to assess for normality given the low number of subjects.

When comparing two groups, data were analysed with a Mann-Whitney test (flow cytometry, ELISA, questionnaire data), a Wilcoxon matched pairs test (stress hormone data), an unpaired t-test (age, BMI data) or a Fisher’s exact test (sex data). When comparing three or more groups, data were analysed with a Kruskal-Wallis test followed by a Dunn’s Multiple comparison test (flow cytometry, ELISA, luminex, questionnaire, BMI data), one-way ANOVA followed by a Bonferroni multiple comparison (age data) or a Chi-square test (sex data). Correlation analyses were performed using the Spearman test. Data given in text, tables and figures are expressed as median followed by range shown as 25^th^ and 75^th^ percentile unless otherwise stated.
